# Treatment Outcome of Severe Acute Malnutrition and Its Determinants among Pediatric Patients in West Ethiopia

**DOI:** 10.1155/2018/8686501

**Published:** 2018-07-30

**Authors:** Muluken Berhanu Mena, Mohammed Gebre Dedefo, Bruke Berhanu Billoro

**Affiliations:** ^1^Bitena Primary Hospital, Wolaita, Ethiopia; ^2^Department of Pharmacy, Wollega University, Nekemte, Ethiopia; ^3^Department of Pharmacy, Wachemo University, Hossana, Ethiopia

## Abstract

**Background:**

Malnutrition is a silent killer that is underreported, underaddressed, and as a result underprioritized. It is reported that severe acute malnutrition is the commonest reason for pediatrics hospital admission in many poor countries; 25 to 30% of children with severe malnutrition die during hospital admissions.

**Objective:**

To determine treatment outcome of severe acute malnutrition and identify its determinants among pediatric patients in pediatrics ward of Nekemte Referral Hospital.

**Methods:**

A retrospective hospital-based cross-sectional study was done from November 2015 to April 2017. Data had been collected by using checklist for recording information from patient card and register book. Association between independent variables and depend variable was assessed using bivariate and stepwise multivariable logistic regression, respectively. Level of statistical significance was declared at p value < 0.05.

**Results:**

Out of 205 admitted children with severe acute malnutrition, 137 (66.8%) cases were cured from SAM, 9 (4.4%) cases were died because of SAM, and (16.6%) cases were defaulter from SAM management, and 25 (12.2%) cases were transferred out. Multivariable logistic regression showed that children admitted with both edema and wasting AOR = 8.30, 95% CI (1.72, 40.09) P=0.008, children without hypothermia AOR = 2.91, 95%CI (1.10, 7.69) P=0.031, children who stay 8-14 days AOR = 3.86, 95%CI (1.01, 14.75) P=0.048, children without pneumonia AOR = 7.82, 95%CI (2.74, 222.29) P=0.001, children without anemia AOR = 3.22, 95%CI (1.04, 9.97) P=0.042, and children without HIV AOR = 9.21, 95% CI (2.20, 38.54) P=0.002 were more likely to be cured from severe acute malnutrition.

**Conclusion:**

Treatment outcome of severe acute malnutrition in this study is good. It shows that around three-fourths of the children were cured. Factors such as admission criteria, hypothermia, length of stay, pneumonia, anemia, and presence of HIV were associated with treatment response.

## 1. Introduction

Malnutrition is a broad term commonly used as an alternative to undernutrition but technically it also refers to overnutrition. In this study the term malnutrition refers to undernutrition. Malnutrition and malnourishment are synonyms of undernutrition and undernourishment, respectively [1]. Severe Acute Malnutrition (SAM) is defined as weight-for-height ratio of less than minus 3 standard deviations below the median reference population or weight-for-height ratio of below 70% or presence of nutritional edema [2].

Malnutrition is a silent killer that is underreported, underaddressed, and as a result underprioritized. Every hour and minute of every day, 300 and 5 children die due to malnutrition respectively. In the world today, one child in four is stunted because of malnutrition, and in underdeveloped countries this figure is as high as one in three and specifically in Africa two out of five children will suffer from malnutrition [3].

Worldwide, it is estimated that there are nearly 20 million children who are severely acutely malnourished. The majority of those affected are found in South Asia and Sub-Sahara Africa. Approximately one million children die every year from severe acute malnutrition [2]. Greater than 25% of children under five in the developing world are undernourished which accounts about 143 million children. Among these 143 million malnourished children, almost three-quarters live in just 10 countries in Sub-Saharan Africa region and more than one-quarter of children under five are undernourished (Nigeria and Ethiopia alone account for more than 33%) [4].

A study conducted in Nigeria showed that 9% of children hospitalized for severe malnutrition were HIV infected [5]. Malnutrition is multifactorial and HIV can induce or aggravate it. In Sub-Saharan Africa, mortality is higher in HIV infected children and ranges from 25% to 38% with severe malnutrition than in noninfected children [5, 6]. During renutrition, mortality was still found to be higher in HIV positive than HIV negative children in Malawi [7, 8].

In a severely malnourished child who has diarrhea, mortality is high ranging from 67.3% to 71% and the cause of death is commonly due to dehydration and electrolytes imbalances. Death is also associated with septicemia, pneumonia, malaria, and hypothermia [6, 9–11].

One way or in another way, malnutrition contributes to 53% of deaths of children under five in developing countries. According to the United Nations International children's Emergency Fund (UNICEF) estimates, around 26 million under-five children suffer from SAM in developing country [12]. Ethiopia is one of the countries with the highest under-five child mortality rate, with malnutrition underlying to 57% of all children deaths [13].

The latest Ethiopia Mini Demography and Health Survey stated that stunting, wasting, and underweight among under-five children in Ethiopia are 40%, 9%, and 25%, respectively, and this figure is very high compared to national figure in Somali region (38.4%, 27.0%, and 38.8%), respectively [14].

This study therefore aimed at determining the treatment outcome and the predictors of poor treatment response among undernourished children admitted to Nekemte Referral Hospital where there is paucity of this important data. The findings of this study intend to further aid clinicians to improve the outcome of these children.

## 2. Methods

### 2.1. Study Setting and Study Period

The study was conducted in Nekemte Referral Hospital (NRH), Nekemte town, Eastern Wollega zone, Oromia National Regional State, which is located at 331 km from Addis Ababa. NRH was established in 1932 (1924 EC) by Swedish mission. Hospital catchment population is 2,028,680. It has different departments and wards like outpatient department (OPD), medical ward, gynecology and obstetrics ward, pediatrics ward, and surgical ward. It delivers diversified health services and clinics including the emergency services, eye clinic, dental clinic, mother child health (MCH), psychiatry clinic, laboratory, radiology, pharmacy, physiotherapy, and followup of chronic diseases. The pediatric ward of NRH had 46 beds. The study was conducted from March 1/2017 to April 1/2017.

### 2.2. Study Design

This study was a retrospective hospital-based cross-sectional study.

### 2.3. Study Population

All malnourished pediatric patients who had been admitted to inpatient pediatric ward of NRH from November 2015 to April 2017 and fulfilled inclusion criteria were included in this study.

### 2.4. Inclusion and Exclusion Criteria

In this study malnourished children with age from 1 month to 14 years and children with a known treatment outcome who were admitted to pediatrics ward were included while children who were discharged within 24 hours and participants with incomplete data were excluded.

### 2.5. Sample Size

Two hundred and five (205) patients diagnosed with severe acute malnutrition and admitted to pediatrics ward from November 2015 to April 2017 and who fulfilled inclusion criteria were studied.

### 2.6. Data Collection Method and Data Quality Control

The data were collected through medical record reviews of pediatric patients, by using a prepared checklist. A checklist addressed all needed information as a data collection tool. Before starting the data collection, data collecting format was cross-matched with available information on records; then the study checklist and weight-for-height chart had been rearranged as necessary. Incomplete chart had been discarded. Data collections were conducted with appropriate training of the data collectors and continuous advices to keep the quality of the data. Close supervision was made by the investigators and the collected data were checked for completeness every day.

### 2.7. Data Processing and Analysis

Data were entered into the Statistical Package for the Social Sciences (SPSS) version 20 for analysis. Both bivariate and multivariate analyses were done by using binary logistic regression. Variables those having association in binary logistic regression was checked by multivariate logistic regression to identify confounders. A bivariate analysis was carried out to see the association between dependent and independent variables. All variables with p value < 0.25 were taken to multivariable model to control all possible confounders odds ratio along with 95% confidence level which was estimated to identify factors associated with the outcome of variable using multivariable logistic regression analysis. Level of statistical significance was declared at p value < 0.05 levels.

### 2.8. Ethics Approval and Consent to Participate

Ethical clearance was obtained from the Ethical Review Committee of Wollega University, College of Health Sciences. This committee has also written a formal letter of permission to Nekemte Referral Hospital to permit accessing the data and cooperate. The Nekemte Referral Hospital also rechecked for ethical compatibility and permitted the data access. As the study was conducted through a review of records, no consent was obtained from the mothers or caregivers of the study subjects. The confidentiality of study participants was secured. In addition all data were kept confidential.

## 3. Results

### 3.1. Sociodemographic and Admission Information

From all admitted children, 138 (67.3%) were under five years of age and 67 (32.7%) of them were between five to fifteen years. From the total children admitted with severe acute malnutrition, 107 (52.2%) were females and 98 (47.8%) were males. Most of the children, 194 (94.6%), were identified as newly admitted children and 11 (5.4%) of them were readmission. Among 205 children, 160 (78.0%) were from rural and most of the admitted children in the program were referred from outpatient department (158) (77.1%). Of the total children, 89 (43.4%) were admitted because of marasmus (wasted) and 36 (17.3%) Kwashiorkor (edematous); 66 (32.2%) were presented with both marasmus and kwashiorkor and 14 (6.8%) of them were admitted because of MUAC (Table 1).

### 3.2. Medical Comorbidities

Of all admitted children, more than three-fourths (3/4) of pediatric patients' appetite at admission was poor (158) (77.1%). The most common medical comorbidities accompanied with SAM children at time of admission were anemia (146) (71.2%), followed by vomiting (82) (40.0%), fever (78) (38.0%), and diarrhea (72) (35.1%) (Table 2).

### 3.3. Treatment Given

Management of children admitted with severe acute malnutrition to pediatrics ward was based on the Federal Ministry of Health of Ethiopia Guideline Protocol for treatment of severe acute malnutrition. Out of 205 children whose medication records were available for review, the most prescribed medications were (137) (66.8%) paracetamol, (114) (55.6%) folic acid, and (110) (53.7%) antibiotic, while the least prescribed was (99) (48.3%) measles vaccine (Table 3).

### 3.4. Treatment Outcome

Out of 205 admitted children with severe acute malnutrition, 137 (66.8%) cases were cured from SAM, 9 (4.4%) cases died because of SAM, 34 (16.6%) cases were defaulter from SAM management, and 25 (12.2%) cases were transferred out from pediatric ward of NRH (Table 4).

### 3.5. Determinants of Treatment Outcome

To know determinants of treatment outcome, the treatment response of the child was classified into two categories as cured and not cured. Not cured includes dyinh and defaulter. Transferring out was excluded because of difficulty to know their treatment outcome. When 25 of the participants were excluded the total sample size to analyze remains 180; from them 137 (76%) were cured and 43(24%) were not cured.

Regarding treatment outcome of each SAM type, for patients with only edema or kwashiorkor of the total 31 patients 20 (64.5%) were cured from SAM, for patients with only wasting or marasmus of the total 80 patients 56 (70%) were cured from SAM, and for patients with both edema and wasting of the total 55 patients 50 (90.9%) were cured from SAM (Table 5).

The bivariable analysis showed that admission criteria, fever, hypothermia, length of stay, pneumonia, anemia, and presence of HIV were associated with treatment response. However, other factors like age, sex, place of residence, weight-for-height, admission type, appetite admission, vomiting, diarrhea, presence of TB, presence of malaria, sever superficial infection, and all routine medications do not show any significant association with treatment response of the child (Table 5).

Variables with P value < 0.25 like age, sex, place of residence, appetite at admission, vomiting, diarrhea, and presence of TB entered multivariable. The results of multivariable logistic regression showed that children admitted with both edema and wasting are 8.3 times more likely to be cured than child admitted by only edema in AOR = 8.30, 95% CI (1.72, 40.09) P=0.008. Children without hypothermia are 2.91 times more likely to be cured than children with hypothermia AOR = 2.91, 95%CI (1.10, 7.69) P=0.031. Children who stay 8-14 days are 3.86 times more likely to recover or cured than children who stay ≤ 7 days AOR = 3.86, 95%CI (1.01, 14.75) P=0.048. Children without pneumonia are 7.82 times more likely to be cured than children with pneumonia in AOR = 7.82, 95%CI (2.74, 222.29) P=0.001. Children without anemia are 3.22 times more likely to be cured AOR = 3.22, 95%CI (1.04, 9.97) P=0.042. The children without HIV were 9.21 times more likely to have positive outcome AOR = 9.21, 95% CI (2.20, 38.54) P=0.002 (Table 5).

## 4. Discussion

The finding of this study showed that, of the study participants with severe acute malnutrition and with known outcome, 76% were cured. Better achievement was recorded in improving treatment outcome as compared to studies conducted in Lusaka, Zambia (53.7%), Kamba, south west Ethiopia (67.7%), Tigray (61.7%), and Jimma ( 45%) [6, 15–17]. However treatment response was poor as compared to similar study conducted from southern Bangladesh (92%), Mardi Niger (91.4%), south Ethiopia (Wolaita (92%), kembata tembaro (92%), and Sidamo zone (94.3%)) [18–22]. In this study achieving low treatment response may be because of inappropriate management of children such as partial prescription of routine medication and due to medical comorbidity at admission such as presence of pneumonia and HIV.

Children admitted with both edema and wasting were identified as positive predictors of treatment response. Similar to this study different study conducted in Harari, Ghana, and Uganda revealed that patients without edema were less likely to recover from SAM [23–25].

The treatment outcome of this study showed that children who stayed 8-14 days (p=0.048) and 15-22 days (p=0.004) had better outcome as compared to children who stayed ≤7 days. Similar to this study a study conducted in Southern Ethiopia [26] reported that children with SAM who stayed longer had achieved recovery criteria.

A study conducted in Tigray health facility reported that the most administered medications were antibiotics (72.13%) and vitamin A (59.17%) while the least was folic acid which was administered to only 5.89% and multivariable analysis showed that taking antibiotics and deworming drugs were positive predictors to recovery rate from SAM. But, having diarrhea, vomiting, and loss of appetite were negative predictors to recovery rate from SAM [15]. However, in this finding the most administered medications were paracetamol (66.8%), folic acid (55.6%), and antibiotics. Different from the study conducted in Tigray [15], the present finding showed that no significant association was seen between treatment outcome and taking antibiotics and deworming. However the present study showed significant association between positive treatment outcome and absence of anemia (p=0.042) and hypothermia (p=0.031).

A study conducted in Yakatit 12 Hospital in Addis Ababa showed that of all the severely malnourished children (24.3%) have pneumonia, (11%) have tuberculosis, and (21%) have diarrhea [27]. Comparable with this, this study showed that from children who were admitted with SAM (21.5%) have pneumonia, (7.8 %) have tuberculosis, and (35.1%) have diarrhea.

The study conducted in Chad on data-analysis revealed that significant associations were found between not cured and diarrhea [7]. Different to this research; the finding of this study did not showed significant association with diarrhea but significant association were seen between high cure rate and absence of HIV (p=0.002) and pneumonia (p=0.001).

This study has its own limitation. The study only used the recorded data of the discharged children to measure treatment outcomes and associated factors. Therefore, this study has limitation in measuring the effect of factors such as education and economic status of parents and completing data on breast feeding. In addition to that there are some factors which had no statistical association with the treatment outcome in this study which may affect the precision. Eventually, users are recommended to take all these into account during interpretation of the findings and degree of precision.

## 5. Conclusion

Treatment outcome of severe acute malnutrition in this study is good. It shows that around thre-fourths of the children were cured. Factors such as admission criteria, hypothermia, length of stay, pneumonia, anemia, and presence of HIV were associated with treatment response. Majority of the admitted children have marasmus type of malnutrition. To improve treatment outcome for children with severe acute malnutrition, continuous supervision has to be done for healthcare professionals to avoid irrational provision of routine medication and attention should be given for improving the capacity of healthcare professionals on proper management of severe acute malnutrition. In addition to this comorbid diseases have to be treated appropriately to increase cure rate.

## Figures and Tables

**Table 1:** Distribution of demographic and admission information on treatment outcome of children with SAM admitted to NRH, West Ethiopia from November 2015 to April 2017 (n=205).

**Variables**	**Categories**	**Frequency**	**Percentages (%)**
Age	1month-1 year	38	18.5
	>1 year-5 years	100	48.8
	>5 years- 10 years	36	17.6
	>10 years-14 years	31	15.1
Sex	Female	107	52.2
	Male	98	47.8
Place of residence	Urban	45	22.0
	Rural	160	78.0
Weight for height or weight for length	<70%	76	37.1
	70-85%	101	49.3
	>85%	28	13.7
MUAC at admission	<110mm	159	77.6
	111-120mm	32	15.6
	>120mm	14	6.8
MUAC at discharge	<110mm	34	16.6
	111-120mm	153	74.6
	>120mm	18	8.8
Admission type	New admission	194	94.6
	Re-admission	11	5.4
Admission criteria	only edema or kwashiorkor	36	17.6
	only wasting or marasmus	89	43.4
	both edema and wasting	66	32.2
	MUAC	14	6.8
Child is referred from	Hospital	32	15.6
	OPD	158	77.1
	Outreach	5	2.4
	SFC	5	2.4
	Spontaneous	5	2.4
Length of stay	≤7 days	23	11.2
	8-14 days	102	49.8
	15-22 days	58	28.3
	≥23 days	22	10.7

**Table 2:** Distribution of medical comorbidities information on treatment outcome of children with SAM admitted to Pediatric Ward of NRH, West Ethiopia, from November 2015 to April 2017 (n=205).

**Variables**	**Frequency**	**Percentages (%)**
Fever	78	38.0
Hypothermia	60	29.3
Appetite at admission		
Good	47	22.9
Poor	158	77.1
Pneumonia	44	21.5
Vomiting	82	40.0
Diarrhea	72	35.1
Types of diarrhea (n=72)		
Watery	66	91.7
Dysentery	6	8.3
Duration of diarrhea (n=72)		
Acute	64	88.9
Persistent	8	11.1
Presence of HIV	21	10.2
Presence of TB	16	7.8
presence of malaria	30	14.6
Anemia	146	71.2
severe superficial Infection	33	16.1

**Table 3:** Distribution of treatment given information on treatment outcome for children with SAM admitted to NRH, West Ethiopia, from November 2015 to April 2017 (n=205).

** Variables**	**Categories**	**Frequency**	**Percentages (%)**
Antibiotic given	Yes	110	53.7
	No	63	30.7
	not indicated	32	15.6
Vitamin A	Yes	102	49.8
	No	57	27.8
	not indicated	46	22.4
measles vaccine	Yes	99	48.3
	No	56	27.3
	not indicated	50	24.4
Fully Immunized	Yes	47	22.9
	No	59	28.8
	not indicated	99	48.3
Folic acid	Yes	114	55.6
	No	27	13.2
	not indicated	64	31.2
Albendazole/Mebendazole	Yes	94	45.9
	No	70	34.1
	not indicated	41	20.0
Paracetamol	Yes	137	66.8
	No	53	25.9
	not indicated	15	7.3

**Table 4:** Distribution of treatment outcome for SAM children who were admitted to NRH, West Ethiopia, from November 2015 to April 2017 (n=205).

**Variables **	**Categories**	**Frequency**	**Percent (%)**
Treatment response of the child	Died	9	4.4
	Cured	137	66.8
	defaulter	34	16.6
	Transferred out	25	12.2
Weight gain for cured in kg (n=137)	Yes	130	94.9
	No	7	5.1
Length of days the child stays for cured (n=137)	≤7 days	4	2.9
	8-14 days	60	43.8
	15-22 days	55	40.1
	≥23 days	18	13.1
MUAC gain for cured in mm (n=137)	Yes	130	94.9
	No	7	5.1

**Table 5:** Results of binary and multivariable logistic regression analysis indicating determinants of treatment outcome of children admitted to pediatric ward of NRH by SAM from November 2015 to April 2017 (n=205).

**Variables**	**Categories**	**Treatment response of the child**		**COR(95%CI)**	**AOR(95%CI) p-value**
		**Not cured (%)**	**Cured (%)**		
Admission criteria	Only edema or kwashiorkor	11(6.1)	20(11.1)	1.00	1.00
	Only wasting or marasmus	24(13.3)	56(31.1)	1.28(0.53,3.08)	1.25(0.34, 4.63)P=0.733
	Both edema and wasting	5(2.8)	50(27.8)	5.50(1.69, 17.85)	8.30(1.72, 40.09)P=0.008
	MUAC	3(1.7)	11(6.1)	2.01(0.46, 8.80)	0.96(0.14, 6.39)P=0.968
Fever	Present	21(11.6)	42(23.3)	1.00	1.00
	Absent	22(12.2)	95(52.8)	2.15(1.07, 4.34)	2.48(0.89, 6.90)P=0.082
Hypothermia	Present	17(9.4)	32(17.8)	1.00	1.00
	Absent	26(14.4)	105(58.3)	2.14(1.03, 4.44)	2.91(1.10, 7.69)P=0.031
Length of stay	≤7 days	12(6.8)	9(5)	1.00	1.00
	8-14 days	19(10.5)	63(35)	4.42(1.61, 12.07)	3.86(1.01, 14.75)P=0.048
	15-22 days	7(3.9)	48(26.7)	9.14(2.82, 29.54)	10.93(2.18, 54.68)P=0.004
	≥23 days	5(2.8)	17(9.4)	4.53(1.21, 16.96)	2.53(0.48, 13.16)P=0.270
Pneumonia	Present	19(10.5)	15(8.3)	1.00	1.00
	Absent	24(13.3)	122(67.7)	6.43(2.87, 14.41)	7.82(2.74, 22.29)P=0.001
Anemia	Yes	34(18.8)	91(50.5)	1.00	1.00
	No	9(5)	46(25.5)	1.91(0.84, 4.31)	3.22(1.04, 9.97)P=0.042
Presence of HIV	Yes	9(5)	9(5)	1.00	1.00
	No	16(8.8)	79(43.8)	4.93(1.69, 14.37)	9.21(2.20, 38.54)P=0.002
	unknown	18(10)	49(27.2)	2.72(0.93, 7.93)	6.92(1.63, 29.27)P=0.009

## Data Availability

The data used to support the findings of this study are available from the corresponding author upon request.
